# Factors Associated with Knowledge of Evacuation Routes and Having an Emergency Backpack in Individuals Affected by a Major Earthquake in Piura, Peru

**DOI:** 10.3390/ijerph192214686

**Published:** 2022-11-09

**Authors:** Mario J. Valladares-Garrido, Luis E. Zapata-Castro, Christopher G. Valdiviezo-Morales, Abigaíl García-Vicente, Darwin A. León-Figueroa, Raúl Calle-Preciado, Virgilio E. Failoc-Rojas, César Johan Pereira-Victorio, Cristian Díaz-Vélez

**Affiliations:** 1 South American Center for Education and Research in Public Health, Universidad Norbert Wiener, Lima 15046, Peru; 2 Oficina de Epidemiología, Hospital Regional Lambayeque, Chiclayo 14012, Peru; 3 Escuela de Medicina, Universidad Nacional de Piura, Piura 20002, Peru; 4 Sociedad Científica de Estudiantes de Medicina de la Universidad Nacional de Piura, Piura 20002, Peru; 5 Emerge, Emerging Diseases and Climate Change Research Unit, School of Public Health and Administration, Universidad Peruana Cayetano Heredia, Lima 15102, Peru; 6 Escuela de Medicina, Universidad de San Martín de Porres, Chiclayo 14012, Peru; 7 Research Unit for Generation and Synthesis Evidence in Health, Universidad San Ignacio de Loyola, Lima 15024, Peru; 8 School of Medicine, Universidad Continental, Lima 15046, Peru; 9 Escuela de Medicina, Universidad Privada Antenor Orrego, Trujillo 13008, Peru; 10 Hospital Nacional Almanzor Aguinaga Asenjo, EsSalud, Chiclayo 14001, Peru

**Keywords:** earthquakes, disaster prevention and mitigation, population education, access to information

## Abstract

Information on the prevention of earthquakes in Peru, a high-risk country, is still emerging. We determined the frequency and factors associated with knowledge of evacuation routes and the use of emergency backpacks in people affected by a major earthquake. A cross-sectional study using secondary data was conducted from August–December 2021 on people that experienced the 6.1 magnitude earthquake that occurred in Piura, Peru on 30 July 2021. The outcome was self-reported knowledge of evacuation routes and the use of emergency backpacks. The association with self-reported earthquake preparation training, use of sources of information on earthquakes, and sociodemographic variables was investigated. A total of 69.5% of participants knew evacuation routes, and 46.3% had an emergency backpack. A higher frequency of knowledge of evacuation routes was associated with previous training (PR: 1.47; 95% CI: 1.15–1.87), use of the media (PR: 1.35; 95% CI: 1.06–1.72), having received information from the COEN (PR: 1.19; 95% CI: 1.02–1.40), and with a greater number of household members (PR: 1.03; 95% CI: 1.01–1.06). There is a high frequency of knowledge of evacuation routes among participants. However, basic notions of prevention culture are still needed. This research contributes to policy development on earthquake preparation at the community level.

## 1. Introduction

Earthquakes are unpredictable natural disasters that have a great impact on people’s health and can also cause material losses [1,2]. It is estimated that in the last two decades, earthquakes have caused almost one million deaths worldwide [2]. Peru is one of the countries at high seismic risk worldwide [3], and its coastal region is at high risk for large-magnitude earthquakes [4]. Community preparation for these events is necessary to respond effectively and mitigate adverse effects [5,6] such as displacement from their homes and the impact on mental and physical health [7]. Being prepared can reduce the number of deaths and injuries after a seismic event; also, having first aid supplies can increase the chances of survival after an earthquake [8]. Previous studies have shown low levels of preparation (12–14%) in families for earthquakes [9,10] and low prevalence (35%) of household evacuation plans [11], as well as both low (0–0.5%) [12,13] and high prevalence (65%) of having emergency kits [14]. For example, 24% of households in a rural community in Trujillo, Peru, had an emergency disaster kit [5].

Exposure to relevant information is a motivator for promoting household emergency preparation [14]. Television and radio are among the main preferred media as a source of communication to prepare people for an earthquake [15], while smartphone applications are considered the main channels of post-earthquake information [16]. In Iran, a study of adult citizens found that women had higher scores for natural disaster practices, while employees had higher knowledge and attitudes [17]. Similarly, higher household income and education have been associated with good household preparedness for public health emergencies such as general disasters and infectious disease outbreaks [18]. In this sense, studies usually have a general approach to natural disaster prevention [5,11,17,18], while those that focus on earthquakes usually only describe variables such as sources of information, level of knowledge, attitudes, or perceptions [9,11,15,16], and some are even conducted on samples of parents, students, older adults, or health personnel [9,10,19,20]; therefore, there is still a lack of information about the culture of earthquake prevention in the general population and its associated factors. Our research question is the following: What are the frequency and factors associated with the knowledge of evacuation routes and having an emergency backpack? The proposed factors to be analyzed here are sex, educational level, type of work, household income, number of family members, sources of information, and earthquake preparedness training. The findings will contribute to the development of policies focused on earthquake preparation, as well as to the elaboration of future research on preventive culture for this type of natural disaster.

Therefore, this study aimed to determine the frequency and factors associated with the self-reported knowledge of earthquake evacuation routes and having an emergency backpack in the population affected by a 6.1 earthquake in Piura, Peru. This paper will describe the study materials and methods (including the study design, population and sample, procedures, instrument, variables, and statistical analysis), results (including the general characteristics of participants, sources of information about earthquakes, the culture of earthquake prevention, factors associated with knowledge of evacuation routes in the event of earthquakes, and factors associated with having an emergency backpack), discussion of main results (including the implications of findings for public health), limitations and strengths of the study, and conclusions.

## 2. Materials and Methods

### 2.1. Study Design

This was a cross-sectional analytical study based on data from a previous study conducted in the resident population of Piura, Peru, which experienced a 6.1 magnitude earthquake on the Richter scale on 30 July 2021 [21,22]. The study used an online survey to collect information on mental health outcomes, sociodemographic data, and earthquake-related information (as explained in the *Procedures* section).

### 2.2. Population and Sample

The population consisted of adults who experienced the earthquake that occurred on 30 July 2021 in Piura and reported being residents of one of the 38 districts of Piura declared in a state of emergency due to the impact of damage caused by the occurrence of seismic movement [23]. The primary study excluded participants who responded with incomplete questionnaires. In the secondary analysis study, we excluded participants who did not respond to the variable of interest (earthquake prevention measures). The sample for this secondary study consisted of the total number of participants registered in the database. Regarding the sample size, we used a confidence level of 95%, a margin of error of 5%, an expected prevalence of 12%, and 10% rejection rate, which resulted in a total of 179 participants in the final sample. The sample for this secondary study consisted of 177 participants registered in the database. The sampling was non-probabilistic snowball sampling.

### 2.3. Procedures

The data were collected and managed using the Research Electronic Data Capture system (REDCap). REDCap is a secure online platform for designing, managing, entering, and rigorously capturing surveys and online databases for research [24,25]. To design the online survey, a template was created in which all the data collection forms were to be included. We clicked on “Add new instrument” and created two forms: (1) informed consent and (2) data collection questionnaires. This process was performed within the Online Designer tool.

Then, we used the Survey Queue tool. This tool allowed us to combine the list of all questionnaires into one single form for each participant. To combine all questionnaires into this single form, we activated the Survey Queue in the REDCap project; we then navigated to “Online Designer” and clicked on the Survey Queue icon located above the data collection instruments. Immediately, a “Set up Survey Queue” box appeared. Next, we clicked the Enable icon for each questionnaire we wanted to set up. Under the “Show survey in survey queue when.” column, we used the drop-down menu to indicate when each questionnaire should be shown to the participant. We used the Branching Logic tool in the Survey Queue to display the questions in the questionnaires. The Branching Logic tool allowed us to display the questionnaires to the participants compiled on a single form automatically, only if the participant provided informed consent.

In addition, we used other tools in the REDCap project to ensure the correct arrangement, provision, and completion of questionnaires: unique and anonymized identifiers on each form, questionnaires ordered in a consistent way ((1) informed consent, (2) general data, (3) sources of information on earthquakes, and (4) culture of earthquake prevention), use of conditional logic for skip questions, mandatory fields in questions to avoid missing, minimum and maximum ranges in numerical variables, and use of group matrix tool for Likert scale responses. Finally, a public survey link was created using the Manage Survey Participants tool. Before starting the study, the survey link and form were verified to work correctly.

Subsequently, we began the dissemination of the virtual questionnaire through infographics on social networks of universities, academic-scientific institutions, and the media in Piura. The questionnaire began its dissemination in August 2021 and ended on 30 September 2021.

### 2.4. Instrument

The questionnaire consisted of 3 sections:

#### 2.4.1. General Data

- Age in years;
- Single marital status (no, yes);
- Level of education (high school, non-university higher education, university higher education);
- Type of work (worker, domestic worker, student, unemployed, other);
- Monthly household income in Peruvian currency (PEN 300 to 1000, PEN 1001 to 2000, PEN 2001 to 3000, PEN 3001 to 5000, and PEN 5001 or more);
- Number of family members in the household, report of having comorbidity (no, yes).

#### 2.4.2. Sources of Information on Earthquakes

- Self-report on the sources of information that the participant used to find out about the earthquake that occurred:
  o Media;
  o Instituto Nacional de Defensa Civil *[National Civil Defense Institute]*—INDECI;
  o Centro de Operaciones de Emergencia Nacional *[National Emergency Operations Center]*—COEN;
  o Sistema Nacional de Gestión de Riesgo de Desastres *[National Disaster Risk Management System]*—SINAGERD;
  o Ministry of Health—MINSA;
  o Instituto Geofísico del Perú *[Geophysical Institute of Peru]*—IGP;
  o Family, colleagues, or friends.

#### 2.4.3. Culture of Earthquake Preparation

- Self-report on having received earthquake preparation training (no, yes);
- Self-report on knowing evacuation routes and safe zones at home and/or workplace (no, yes);
- Self-report on having an emergency backpack for earthquakes (no, yes).

### 2.5. Variables

The dependent variables were (1) report knowing evacuation routes and safe zones in their home and/or workplace through the question “Do you know evacuation routes and safe zones in your home and/or workplace?” and (2) report having an emergency backpack for earthquakes with the question “Do you have an emergency backpack for earthquakes”?

The independent variables were report having received earthquake preparation training with the question “Have you received earthquake preparation training?”, use of information sources used to learn about the earthquake, and sociodemographic data (age, marital status, level of education, type of work, monthly household income, number of family members, and report of having any comorbidity).

### 2.6. Statistical Analysis

Statistical analysis was performed in Stata 17.0 (StataCorp, College Station, TX, USA, 2016) after exporting the database from the REDCap data entry system.

In the descriptive analysis, we reported absolute and relative frequencies for categorical variables and the best measure of central tendency and dispersion for numerical variables, previously evaluating the normal distribution numerically, graphically, and analytically.

In the bivariate analysis, we identified the factors associated with knowledge of earthquake evacuation routes and having an earthquake emergency backpack using the chi-square test of independence after analyzing the expected frequency assumption. In the case of numerical variables, the parametric Student’s *t*-test was useful; otherwise, the nonparametric Mann–Whitney U test was used.

In simple and multiple regression, we estimated prevalence ratios (PR) and 95% confidence intervals (95% CI). We used generalized linear models (GLM), *Poisson* distribution family, log link function, and *cluster* by the district of origin. Significant variables (*p* < 0.05) in the simple model entered the final multiple-variable model. *p*-values less than 0.05 were reported as statistically significant.

## 3. Results

### 3.1. General Characteristics of Participants

Of the 177 participants, the mean age was 22 years, 56% were female, 63.8% had a university education, and 28.3% were working at the time of their participation; Table 1.

### 3.2. Sources of Information about Earthquakes

**Figure 1:**
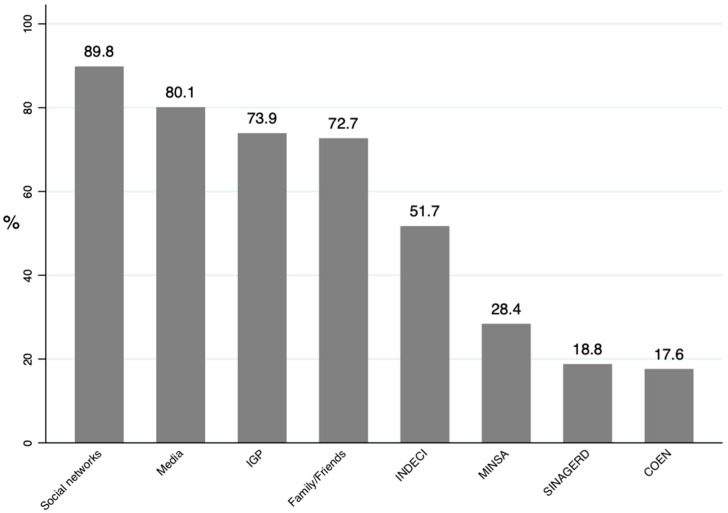
Source of seismic information reported by participants.

We found that 89.8% of the participants reported using social networks to receive information about the earthquake. In addition, the other most frequently reported sources of information were the media (80.1%), the Geophysical Institute of Peru-IGP (73.9%), and the National Institute of Civil Defense-INDECI (51.7%). Only 17.6% and 18.8% of the participants reported using the National Emergency Operations Center (COEN) and the National Disaster Risk Management System (SINAGERD) as a source of information about the earthquake; Figure 1.

### 3.3. Culture of Earthquake Prevention

Here, 63.3% reported having received earthquake preparation training, while 69.5% mentioned knowing evacuation routes and safe zones in their home and/or workplace. In addition, slightly less than half (46.3%) mentioned having an earthquake emergency backpack at home; Table 1.

### 3.4. Factors Associated with Knowledge of Evacuation Routes in the Event of Earthquakes

In the bivariate analysis, we found that the variables significantly associated with knowledge of earthquake evacuation routes were using the media (*p* = 0.003), IGP (*p* = 0.029), and family (*p* = 0.001) as sources of information for earthquakes. In addition, participants who reported having received earthquake preparation training had a higher frequency of knowing evacuation routes (80.4% vs. 50.8%; *p* < 0.001); Table 2.

In the multiple regression analysis (adjusted for current job, family members in the household, comorbidity, sources of information, and earthquake preparation training), the factors associated with a higher frequency of knowledge of earthquake evacuation routes were reporting having received earthquake preparation training (PR: 1.47; 95% CI: 1.15–1. 87), using the media as a source of information about the earthquake (PR: 1.35; 95% CI: 1.06–1.72), getting information through the COEN (PR: 1.19; 95% CI: 1.02–1.40), and having more members in the household (PR: 1.03; 95% CI: 1.01–1.06). On the contrary, being a student reduces by 18% the frequency of knowing evacuation routes and safe zones in their home or workplace (PR: 0.82; 95% CI: 0.69–0.98); Table 3.

### 3.5. Factors Associated with Having an Emergency Backpack for Earthquakes

The factors associated with having an emergency backpack were reporting the MINSA as a source of earthquake information (PR: 1.24; 95% CI: 1.03–1.48) and having a family income greater than PEN 3000 (PR: 1.58; 95% CI: 1.15–2.18), as found in the multiple regression analysis adjusted for household income and MINSA as a source of information (the only two significant variables in the simple regression model); Table 3.

## 4. Discussion

Earthquakes, one of the most damaging natural disasters, leave a large number of people injured, disabled, or dead [26]. Taking scientific and reasonable earthquake preparedness measures can effectively reduce the casualties and economic losses caused by earthquakes [27]. In this research, we determined the frequency and factors associated with self-reported knowledge of earthquake evacuation routes and having an emergency backpack in the population affected by the earthquake in Piura, Peru.

### 4.1. Sources of Information about Earthquakes

In our research, it was found that the most used source of information to be informed about earthquake news was social networks (89.8%), mass media (80.1%), the Geophysical Institute of Peru-IGP (73.9%), and the National Institute of Civil Defense-INDECI (51.7%). In contrast, the least reported sources of information were the National Disaster Risk Management System-SINAGERD (18.8%) and COEN (17.6%). Similar to this was the study after the 2011 earthquake in Japan (8.9°), in which the population used social networks, especially Twitter, and traditional media such as television and newspapers to stay informed [28]. However, a review on the use of technology during natural disasters reported that there are still limitations regarding social networks due to the reliability of the information shared and its poor accessibility in vulnerable populations such as the elderly [29]. The use of social networks as the main source of information is related to their great usefulness for dissemination, allowing response, and alerting in emergencies [30].

### 4.2. Culture of Earthquake Prevention

We found that nearly 6 out of 10 participants reported having received earthquake preparation training. This is similar to that reported by Ozdemir, Raziye et al. in Turkey, where 61.4% of Karabuk University faculty members reported reading tutorials on earthquake preparation, and 47.7% received first aid training [8]. However, it is higher than what was found by another study conducted on Turkish physicians, in which more than 50% of the respondents stated that they had never attended a disaster drill [31]. This finding could be explained by the fact that the frequency of earthquake training is moderately acceptable [12] and depends on the level of education, economy, and experience that people have in dealing with earthquakes [26].

Nearly 7 out of 10 participants reported knowing evacuation routes and safe zones in their homes and/or workplaces. This is similar to that reported by Gallegos C. R. in students in Lima, 74% knew about protective places inside the classroom, and 65% [32] about evacuation routes [33]. However, this is lower than that found in American Samoa, where 35% (88/251) of the participating households did not have an evacuation plan [11]. This finding could be explained by the level of knowledge of preventive measures in case of an earthquake and the probability of a potentially damaging earthquake.

Our study found that nearly 4 out of 10 participants have an emergency backpack in the event of an earthquake. This is similar to that reported in the 2021 National Household Survey in the United States, where 45% reported preparing supplies [34]. However, it is lower than that reported by Stewart et al. in Trujillo, Peru, where approximately 24% of participants had an earthquake backpack [5]. This differs even more from a study from South Delhi, India, where 99% of the respondents had never heard of any disaster kit [12]. This finding could be explained by a difference between the socioeconomic level of the populations studied and the inadequate preparation and perception of the risk that can be caused by earthquakes [14].

### 4.3. Factors Associated with Knowledge of Evacuation Routes in the Event of Earthquakes

People who reported having received earthquake preparation training were 47% more likely to know evacuation routes and safe zones in their homes or workplaces. This is similar to that reported by a quasi-experimental study in Turkey, where students who received an educational intervention had a 48.5% increase in determining how to leave their homes during an earthquake [9]. A study of nurses in Bangladesh found that those who received training were more prepared for disasters [35]. This finding could be explained by the fact that through training, people have access to education and training to respond to emergencies [19].

Using the media as a source of information about the earthquake increased 53% the frequency of knowing evacuation routes and safe zones in their home or workplace. This is similar to what was reported in Indonesia, where the media, such as television and radio, proved to be effective in transmitting information about the earthquakes that occurred [36]. This finding could be explained by the fact that the media can be channels for earthquake preparation in a reliable manner, with credible information and high visibility in the community [37,38].

People who were informed through the COEN are 20% more likely to know evacuation routes and safe zones in their homes or workplace. A study conducted in Hong Kong found that only 2.9% of participants used state websites as a source of disaster information [37]. Another study conducted in Asia found that approximately 80% of participants in Vietnam considered that the information provided by the government for disaster prevention could be trusted [39]. This finding could be explained by the fact that the COEN provides validated and timely information on disasters for use by both authorities and the population [40].

The greater the number of household members, the greater the frequency of knowing evacuation routes and safe zones in their homes or workplaces. This is similar to what was reported in Hong Kong, where a higher number of household members was associated with good household disaster preparation [18]. However, this differs from that reported in Iran, where households with four or fewer members are associated with higher disaster preparation and social trust [41]. A study of a sample of older adults found that the composition of their households was not related to knowledge of evacuation routes [20]. This finding could be explained by the influence of family members as a social group so that the number of members in the household would involve sharing a greater amount of information about earthquake preparation through family discussions [38].

Students were 18% less likely to know evacuation routes and safe zones in their homes or workplace. This is similar to what was reported in Iran, where less knowledge about disaster preparation was found in students and self-employed workers [17], and another study conducted in the same country found less preparation for earthquakes in people with pre-university education [26]. Nevertheless, this differs from what was found in American Samoa, where no relationship was found between higher education and having an evacuation plan for earthquakes and tsunamis [11]. This finding could be explained by the lack of disaster preparation training in universities, despite being located in a northern Peruvian city historically affected by the Coastal El Niño phenomenon and the probable occurrence of earthquakes due to its location in the Pacific Ring of Fire. Courses or training are given superficially because they lack a focus associated with skills that are attractive to students, such as first aid, rescue skills, or simulated disaster scenarios [42].

### 4.4. Factors Associated with Having an Emergency Backpack for Earthquakes

We found that people who used the Ministry of Health (MINSA) as a source of earthquake information had a 24% higher frequency of having an emergency backpack. Moran Bodas et al. in their study reported that the frequency of searching for information on earthquakes represented 79.1% [14] and according to Matthew Stewart et al., 26% had an emergency food supply, 24% had an emergency water supply, 24% had a first aid kit, and only 20% had a family evacuation plan [5]. According to the National Institute of Civil Defense (INDECI), it recommends that Peruvians have a “Survival Combo”, which consists of an emergency backpack and a reserve box, which every family should have on hand in case of a strong earthquake [43]. This is similar to that reported by Burke Sloane et al., who reported that the Red Cross (67%), police (65%), and firefighters (65%) were the most cited sources from which workers could receive emergency information in the event of a natural disaster [44]. This could be explained by the fact that those who receive information from MINSA sources report a higher frequency of having an emergency backpack; probably, the fact of receiving valid and reliable information from the Peruvian Ministry of Health influences attitudes toward earthquake prevention. In addition, MINSA proposes health policies for the prevention and care of emergencies and disasters, seeking to ensure that the population is prepared and knows what measures to adopt in the event of a major earthquake [45].

In addition, those with a family income of more than PEN 3000 were 58% more likely to have an emergency backpack. This is similar to that reported in Taiwanese families by Ziqiang Han et al., in which the frequency of having an emergency preparation backpack is higher in families with higher average monthly household income (TWD 70,000 and 89,999; 19% vs. 13%; TWD 60,000 and 79,000) [46]. This finding could be explained by the relationship between educational level and economic level, which would generate earthquake prevention behaviors.

### 4.5. Implications of Findings for Public Health

Through this research, we have studied the factors associated with the culture of prevention in risk situations such as earthquakes. Our findings indicate that more than half of the population knew the evacuation routes and safe zones (69.49%), which was related to having received previous training [9] and using the media to become informed [36,47]. Given this situation, we consider it important to continue educational programs aimed at the population, especially among students who reported less preparation [17] and to implement interactive technological programs [48].

Training should be considered among the main strategies for the mitigation of damage caused by natural disasters according to their magnitude [32], although there are already some online courses on knowledge and risk management available on the website of the National Institute of Civil Defense (INDECI) [49], it is necessary to promote their dissemination through the media and social networks, and also to carry out face-to-face and virtual interventions to orient the population.

In our study, approximately half of the participants did not have an emergency backpack (53.67%), as recommended by the Ministry of Health (MINSA) [50]; therefore, it is important to analyze the viability of the preventive measures proposed at the national level since many families have difficulty accessing the supplies included in these kits. Therefore, it is important to analyze the viability of the preventive measures proposed at the national level since many families have difficulty accessing the supplies included in these kits [51] and live in areas vulnerable to earthquakes, such as the coastal zone [4].

Concerning the health problems framed by the study, it is important to understand that the effects of the earthquake on public health began to be studied both in the early stages and in terms of medium–long-term effects [52]. These natural phenomena have an impact on people’s lives and cause deaths and severe injuries [53]. In addition, exposure to pollutants and the environment had a clear impact on public health. Acute health effects of air pollution can include the development of cardiovascular and respiratory disorders, acute respiratory infections, and bronchial asthma attacks; these effects are mainly caused by oxidative stress and inflammation [52]. Several mental health problems were also mentioned, such as psychological distress and post-traumatic stress disorder [54]. It is therefore important to study the potential medium- and long-term health impacts of all upcoming earthquakes. Because earthquakes have been associated with a range of adverse health outcomes, careful monitoring of their long-term health consequences is essential for prioritizing regional and global public health initiatives [55].

## 5. Limitations and Strengths

Our research has some limitations. First, there is a selection bias since we conducted a non-probabilistic sampling; therefore, it is not possible to generalize findings to the population of interest. Second, due to the cross-sectional design, it is not possible to attribute causality between the variables that were associated with a better culture of earthquake prevention. Third, there could be an information bias because the variables were measured by self-reporting of the participants; however, to reduce this, we collected information up to a maximum of two months after the earthquake. However, our study highlights as its main strength the fact that the results found are consistent with the literature and with earthquake preparation, a crucial stage in natural disasters to mitigate possible aftereffects as much as possible. In addition, our research addresses an issue that is scarcely studied in northern Peru. Finally, this study shows solid and well-documented evidence to prepare better community policies that are efficient, culturally adapted to our context, and serve as a niche for future research aimed at strengthening the preventive culture in the face of natural disasters.

## 6. Conclusions

We found a high frequency of knowledge about evacuation routes and safe zones at home or workplace in the participants exposed to the strong earthquake. However, slightly less than half reported having an emergency backpack in the event of an earthquake. In addition, the factors associated with a better culture of earthquake prevention were being informed of the earthquake by the media, COEN, and family. Participants with higher income and those who were informed about the earthquake by MINSA reported a higher frequency of having an emergency backpack in the event of an earthquake. These findings suggest that there is still a need to increase awareness of the culture of earthquake prevention and that this depends on focused interventions by authorities, in which the participation of institutions and media may play a fundamental role. Our study will contribute to the development of policies focused on earthquake preparation, as well as to the elaboration of future research on preventive culture in the face of natural disasters and their impact on different aspects of health. Recommendations for future studies comprise the inclusion of a random sample, the development of cohort studies that measure the actual practice of evacuation routes and the use of emergency backpacks, and the establishment of interventions that effectively promote these practices.

It is recommended to design and implement interventions to improve earthquake preparedness based on self-efficacy, perceived benefits, and action cues. Planning, preparing, and practicing what you would do in the event of an earthquake will help you learn to react appropriately and naturally when the shaking begins. This will increase the chances of surviving an earthquake and reduce the danger of injury. 

In addition, it is vital to stock up on emergency supplies that can be used after an earthquake, including a first aid kit and emergency supply kits for the house and car with water and food, and stocking up on products that will last at least three days. In addition, you should gather necessary documentation and compile a list of crucial details, such as emergency contact information and relevant medical information (such as medical documents, birth certificates, and passports), and store these items in a safe place, such as a fireproof or airtight safe.

## Figures and Tables

**Table 1:** Characteristics of participants (n = 177).

Characteristics		N (%)
Age (years) *		22 (20–29)
Gender		
	Female	98 (56.0)
	Male	77 (44.0)
Single		
	No	37 (20.9)
	Yes	140 (79.1)
Level of education		
	Secondary	46 (26.0)
	Higher non-university	18 (10.2)
	Higher university	113 (63.8)
Current job		
	Employer	50 (28.3)
	Home worker	9 (5.1)
	Student	98 (55.4)
	Unemployed	7 (4.0)
	Others	13 (7.3)
Household income in Soles		
	PEN 300 to 1000	28 (15.8)
	PEN 1001 to 2000	65 (36.7)
	PEN 2001 to 3000	22 (12.4)
	PEN 3001 to 5000	31 (17.5)
	PEN 5001 or more	31 (17.5)
Family members in the household *		4 (3–5)
Comorbidity		
	No	147 (84.5)
	Yes	27 (15.5)
Earthquake preparation training		
	No	65 (36.7)
	Yes	112 (63.3)
Knowledge of evacuation routes and safe zones at home or workplace		
	No	54 (30.5)
	Yes	123 (69.5)
Have an emergency backpack for earthquakes		
	No	95 (53.7)
	Yes	82 (46.3)

* Median (25th percentile–75th percentile); some variables do not sum up to 177 due to missing data.

**Table 2:** Factors associated with knowledge about evacuation routes and having an emergency backpack in case of earthquakes.

Variables		*Knowledge of Evacuation Routes and Safe Zones*		p *	*Have an Emergency* *Backpack for Earthquakes*		p *
		No (n = 54)	Yes (n = 123)		No (n = 95)	Yes (n = 82)	
		n (%)	n (%)		n (%)	n (%)	
Age (years) ***		22 (20–29)	22 (20–29)	0.778 **	22 (20–29)	22 (20–29)	0.887 **
Gender				0.937			0.526
	Female	30 (30.6)	68 (69.4)		50 (51.0)	48 (49.0)	
	Male	24 (31.2)	53 (68.8)		43 (55.8)	34 (44.2)	
Single				0.358			0.427
	No	9 (24.3)	28 (75.7)		22 (59.5)	15 (40.5)	
	Yes	45 (32.1)	95 (67.9)		73 (52.1)	67 (47.9)	
Level of education				0.528			0.721
	Secondary	11 (23.9)	35 (76.1)		23 (50.0)	23 (50.0)	
	Higher non-university	6 (33.3)	12 (66.7)		11 (61.1)	7 (38.9)	
	Higher university	37 (32.7)	76 (67.3)		61 (54.0)	52 (46.0)	
Current job				0.062			0.607
	Employer	10 (20.0)	40 (80.0)		25 (50.0)	25 (50.0)	
	Home worker	6 (66.7)	3 (33.3)		7 (77.8)	2 (22.2)	
	Student	33 (33.7)	65 (66.3)		53 (54.1)	45 (45.9)	
	Unemployed	2 (28.6)	5 (71.4)		4 (57.1)	3 (42.9)	
	Others	3 (23.1)	10 (76.9)		6 (46.2)	7 (53.9)	
Household income in Soles				0.525			0.294
	PEN 300 to 1000	9 (32.1)	19 (67.9)		17 (60.7)	11 (39.3)	
	PEN 1001 to 2000	18 (27.7)	47 (72.3)		35 (53.9)	30 (46.2)	
	PEN 2001 to 3000	4 (18.2)	18 (81.8)		11 (50.0)	11 (50.0)	
	PEN 3001 to 5000	12 (38.7)	19 (61.3)		12 (38.7)	19 (61.3)	
	PEN 5001 or more	11 (35.5)	20 (64.5)		20 (64.5)	11 (35.5)	
Family members in the household †		4.26 ± 4.0	4.79 ± 5.0	0.089 ¶	4.57 ± 1.97	4.7 ± 1.75	0.654 ¶
Comorbidity				0.312			0.761
	No	47 (32.0)	100 (68.0)		77 (52.4)	70 (47.6)	
	Yes	6 (22.2)	21 (77.8)		15 (55.6)	12 (44.4)	
Sources of information							
	Media	36 (25.5)	105 (74.5)	**0.003**	75 (53.2)	66 (46.8)	0.908
	Social networks	47 (29.6)	112 (70.4)	0.415	85 (53.5)	74 (46.5)	0.866
	INDECI	25 (27.5)	66 (72.5)	0.339	43 (47.3)	48 (52.8)	0.09
	COEN	5 (16.1)	26 (83.9)	0.053	13 (41.9)	18 (58.1)	0.158
	SINAGERD	6 (18.2)	27 (81.8)	0.084	14 (42.4)	19 (57.6)	0.16
	MINSA	12 (24.0)	38 (76.0)	0.226	23 (46.0)	27 (54.0)	0.215
	IGP	34 (26.2)	96 (73.9)	**0.029**	65 (50.0)	65 (65.0)	0.127
	Family, friends	27 (21.1)	101 (78.9)	**<0.001**	67 (52.3)	61 (47.7)	0.644
Earthquake preparation training				**<0.001**			0.056
	No	32 (49.2)	33 (50.8)		41 (63.1)	24 (36.9)	
	Yes	22 (19.6)	90 (80.4)		54 (48.2)	58 (51.8)	

* *p*-value for categorical variables were calculated with the chi-square test; ** *p*-value for age was calculated with the U-test (Mann–Whitney); *** median—interquartile range; † mean ± standard deviation; ¶ *p*-value for family members in the household was calculated with the Student’s *t*-test; some variables do not sum up to 177 due to missing data.

**Table 3:** Factors associated with knowledge about evacuation routes and having an emergency backpack in case of earthquakes, in simple and multiple regression analysis.

Characteristics		*Knowledge of Evacuation Routes and Safe Zones*						*Have an Emergency Backpack for Earthquakes*					
		Simple Regression			Multiple Regression			Simple Regression			Multiple Regression		
		PR	CI 95%	p *	PR	CI 95%	p *	PR	CI 95%	p *	PR	CI 95%	p *
Age (years) *		1.00	0.99–1.01	0.076				1.00	0.99–1.01	0.897			
Gender													
	Female	Ref.						Ref.					
	Male	1.00	0.87–1.14	0.908				0.90	0.68–1.20	0.481			
Single													
	No	Ref.						Ref.					
	Yes	0.90	0.79–1.02	0.092				1.18	0.76–1.83	0.456			
Level of education													
	Secondary	Ref.						Ref.					
	Higher non-university	0.88	0.67–1.15	0.337				0.78	0.38–1.57	0.485			
	Higher university	0.88	0.66–1.19	0.419				0.92	0.73–1.16	0.475			
Current job													
	Employer	Ref.			Ref.			Ref.					
	Home worker	0.42	0.20–0.86	**0.017**	0.44	0.17–1.10	0.078	0.44	0.18–1.12	0.085			
	Student	0.83	0.71–0.97	**0.016**	0.82	0.69–0.98	**0.025**	0.92	0.64–1.32	0.643			
	Unemployed	0.89	0.69–1.15	0.381	0.85	0.66–1.11	0.239	0.86	0.49–1.51	0.592			
	Others	0.96	0.66–1.40	0.837	0.91	0.66–1.26	0.570	1.08	0.57–2.05	0.821			
Household income in Soles													
	PEN 300 to 1000	Ref.						Ref.			Ref.		
	PEN 1001 to 2000	1.07	0.87–1.31	0.545				1.17	0.79–1.75	0.425	1.21	0.80–1.84	0.374
	PEN 2001 to 3000	1.21	0.96–1.51	0.102				1.27	0.86–1.88	0.226	1.31	0.90–1.90	0.156
	PEN 3001 to 5000	0.90	0.82–1.00	0.050				1.56	1.17–2.08	**0.003**	1.58	1.15–2.18	**0.005**
	PEN 5001 or more	0.95	0.67–1.36	0.781				0.90	0.42–1.94	0.794	0.93	0.42–2.06	0.858
Family members in household *		1.05	1.01–1.08	**0.013**	1.03	1.01–1.06	**0.015**	1.02	0.96–1.08	0.546			
Comorbidity													
	No	Ref.			Ref.			Ref.					
	Yes	1.14	1.01–1.29	**0.032**	1.11	0.95–1.29	0.176	0.93	0.80–1.08	0.361			
Sources of information													
	Media	1.53	1.21–1.94	**<0.001**	1.35	1.06–1.72	**0.017**	1.02	0.84–1.24	0.812			
	Social networks	1.15	0.78–1.71	0.480				1.05	0.82–1.34	0.714			
	INDECI	1.10	0.88–1.38	0.407				1.32	0.83–2.09	0.238			
	COEN	1.27	1.13–1.42	**<0.001**	1.19	1.02–1.40	**0.025**	1.32	0.92–1.87	0.128			
	SINAGERD	1.23	1.06–1.43	**0.007**	1.02	0.84–1.24	0.826	1.31	0.85–2.00	0.217			
	MINSA	1.14	1.04–1.25	**0.004**	0.93	0.81–1.06	0.285	1.24	1.03–1.49	**0.023**	1.24	1.03–1.48	**0.021**
	IGP	1.31	1.02–1.67	**0.032**	0.98	0.71–1.35	0.901	1.35	0.95–1.93	0.097			
	Family, friends	1.80	1.45–2.24	**<0.001**	1.53	1.22–1.90	**<0.001**	1.09	0.85–1.39	0.494			
Earthquake preparation training													
	No	Ref.			Ref.			Ref.					
	Yes	1.58	1.31–1.92	**<0.001**	1.47	1.15–1.87	**0.002**	1.40	0.92–2.14	0.118			

* *p*-values obtained with Generalized Linear Models (GLM), Poisson family, log-link function, robust variance, cluster by the district.

## Data Availability

The dataset generated and analyzed during the current study is not publicly available because the ethics committee has not provided permission/authorization to publicly share the data but are available from the corresponding author on reasonable request.
